# Strongly Coupled Magnetic and Electronic Transitions in Multivalent Strontium Cobaltites

**DOI:** 10.1038/s41598-017-16246-z

**Published:** 2017-11-22

**Authors:** J. H. Lee, Woo Seok Choi, H. Jeen, H.-J. Lee, J. H. Seo, J. Nam, M. S. Yeom, H. N. Lee

**Affiliations:** 1 School of Energy and Chemical Engineering, Ulsan National Institute of Science & Technology (UNIST), Ulsan, 44919 Republic of Korea; 2 Materials Science and Technology Division, Oak Ridge National Laboratory, Oak Ridge, TN 37831 USA; 3 Department of Physics, Sungkyunkwan University, Suwon, 440-746 Korea; 4 Department of Physics, Pusan National University, Busan, 46241 Korea; 5 Department of Applied Research and Network R&D, Center for Computational Science and Engineering, Division of National Supercomputing R&D, Korea Institute of Science and Technology Information (KISTI), 245 Daehak-ro, Daejeon, 34141 Republic of Korea

## Abstract

The topotactic phase transition in SrCoO_*x*_ (*x* = 2.5–3.0) makes it possible to reversibly transit between the two distinct phases, i.e. the brownmillerite SrCoO_2.5_ that is a room-temperature antiferromagnetic insulator (AFM-I) and the perovskite SrCoO_3_ that is a ferromagnetic metal (FM-M), owing to their multiple valence states. For the intermediate *x* values, the two distinct phases are expected to strongly compete with each other. With oxidation of SrCoO_2.5_, however, it has been conjectured that the magnetic transition is decoupled to the electronic phase transition, i.e., the AFM-to-FM transition occurs before the insulator-to-metal transition (IMT), which is still controversial. Here, we bridge the gap between the two-phase transitions by density-functional theory calculations combined with optical spectroscopy. We confirm that the IMT actually occurs concomitantly with the FM transition near the oxygen content *x* = 2.75. Strong charge-spin coupling drives the concurrent IMT and AFM-to-FM transition, which fosters the near room-*T* magnetic transition characteristic. Ultimately, our study demonstrates that SrCoO_*x*_ is an intriguingly rare candidate for inducing coupled magnetic and electronic transition via fast and reversible redox reactions.

## Introduction

Coupling between magnetism and charge (or electricity) has triggered many fascinating phenomena, including colossal magnetoresistance^1–3^ and magnetoelectric effect^4,5^. It has been known that the strong competition between the antiferromagnetic super-exchange mechanism^6^ and the Zener double-exchange mechanism^7^ triggered by both doping and epitaxial strain brings out intriguing magnetoelectric phenomena and subsequent exotic phases.

Oxygen stoichiometry in the transition metal oxides (TMOs) plays an essential role in determining the physical properties, including optoelectronic and magnetic properties^8–10^. The multivalent nature of most transition metals often causes the formation of various solid TMO phases with different oxidation states, yielding intriguing oxygen concentration-dependent electronic and magnetic phase diagrams^11–13^. The oxygen content and consequent valence state of transition metals are also closely related to the ionic conduction and catalytic activities, which are critical in most cutting-edge energy storage and generation devices^14–17^. Therefore, exploring the role of oxygen defects in determining the electronic and magnetic properties would provide insight into identifying new possibilities for TMOs as energy materials.

Among TMOs, SrCoO_*x*_ (SCO, 2.5 ≤ *x* ≤ 3.0) is an excellent candidate for studying the oxygen-content driven coupled phase transitions. Unlike LaCoO_3_, which shows good oxygen stability due to the robust Co^3+^ valence state^18^, SCO undergoes oxygen-content-dependent topotactic phase transitions. The latter accompany gigantic modifications in both its electronic and magnetic structures owing to the low oxygen sublattice stabilty^12,19–21^. It is worth mentioning that the brownmillerite SCO (*x* = 2.5, BM-SCO) exhibits a G-type AFM insulating phase with a high Neel temperature (*T*_N_ = 540 K) and, the perovskite SCO (*x* = ~3.0, P-SCO), which is a ferromagnetic metal, exhibits one of the highest Curie temperatures (*T*_C_ = 305 K) among 3*d* transition metal oxides. However, due to the difficulty in synthesizing single phase perovskites, the coupling of the magnetic transition to the insulator-to-metal transition (IMT) and its abruptness have not been much explored, whereas a lot is known for other TMOs such as manganites^3^. While the AFM in BM-SCO originates from superexchange in the insulating phase, the ferromagnetism in P-SCO originates from a distinct mechanism with itinerant holes mediating local Co spins^22^. Also, a study using neutron and x-ray scattering reported an abrupt transition in charge status from Co^3+^ to Co^4+^ in SCO at *x* = ~2.82 and in structure from orthorhombic to cubic at *x* = ~2.75^23^. Therefore, understanding the microscopic picture of how the extreme phases (AFM-I vs FM-M) can be transformed via the reversible redox reactions^13^ is important both in fundamentals and new potentials of these materials.

Here, we present a combined study of density-functional theory (DFT) calculations and optical spectroscopy to reveal the coupling between itinerancy of charge carriers and magnetic transition mediated by the oxygen concentration in SCO_*x*_. The DFT results predict that charge-spin coupling is sufficiently strong that the IMT is triggered by the magnetic transition. It is also found that the Drude peak in optical spectroscopy (indication of metallicity) occurs concomitantly with the formation of strong sigma bonding, facilitating the itinerant hole conduction and the stabilization of consequent ferromagnetism. Thus, we conclude that the strongly coupled nature may be responsible for the abrupt transition from AFM to FM.

## Result

### IMT with oxidation revealed by optical conductivity

**Figure 1:**
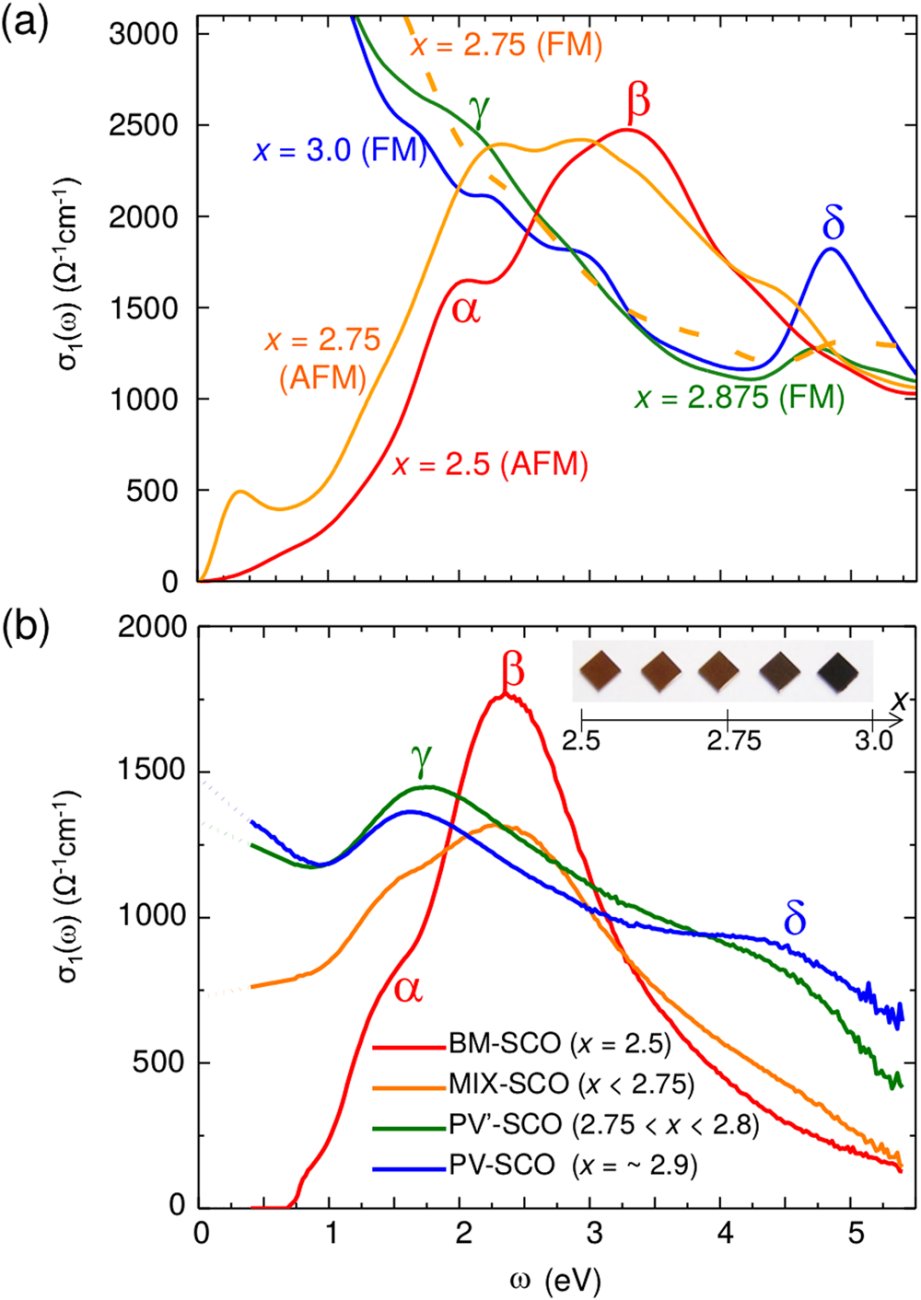
Evolution of optical conductivity. (**a**) Calculated optical conductivity spectra (*σ*_1_(*ω*)) from DFT calculations for *x* = 2.5, 2.75, 2.875, and 3.0 in their magnetic ground states (bold lines). FM and AFM denote ferromagnetic and G-type antiferromagnetic ground states, respectively. For *x* = 2.75, we additionally computed *σ*_1_(*ω*) for a ferromagnetic state (*x* = 2.75(FM)), which is shown with a dotted line, for comparison with the G-AFM ground state marked with *x* = 2.75(AFM). (**b**) Experimental *σ*_1_(*ω*) for SCO thin films with various oxidation states. With increasing *x*, the Drude peak evolves with the emergence of *δ* peak. The inset shows photographic images of SCO thin films, which show a systematic color change with *x*. “BM” and “PV” denote Brownmillerite and perovskite phases, respectively. “MIX” denotes BM rich composition when *x* is smaller than 2.75 and “PV′” denotes PV perovskite rich phase when *x* is larger than 2.75. “BM-SCO”, “MIX-SCO”, “PV′-SCO”, and “PV-SCO” are represented with red, orange, green, and blue line, respectively. *α*, *β*, *γ*, and *δ* are representative peaks for comparison between theoretical (**a**) and experimental (**a**) conductivities.

**Figure 2:**
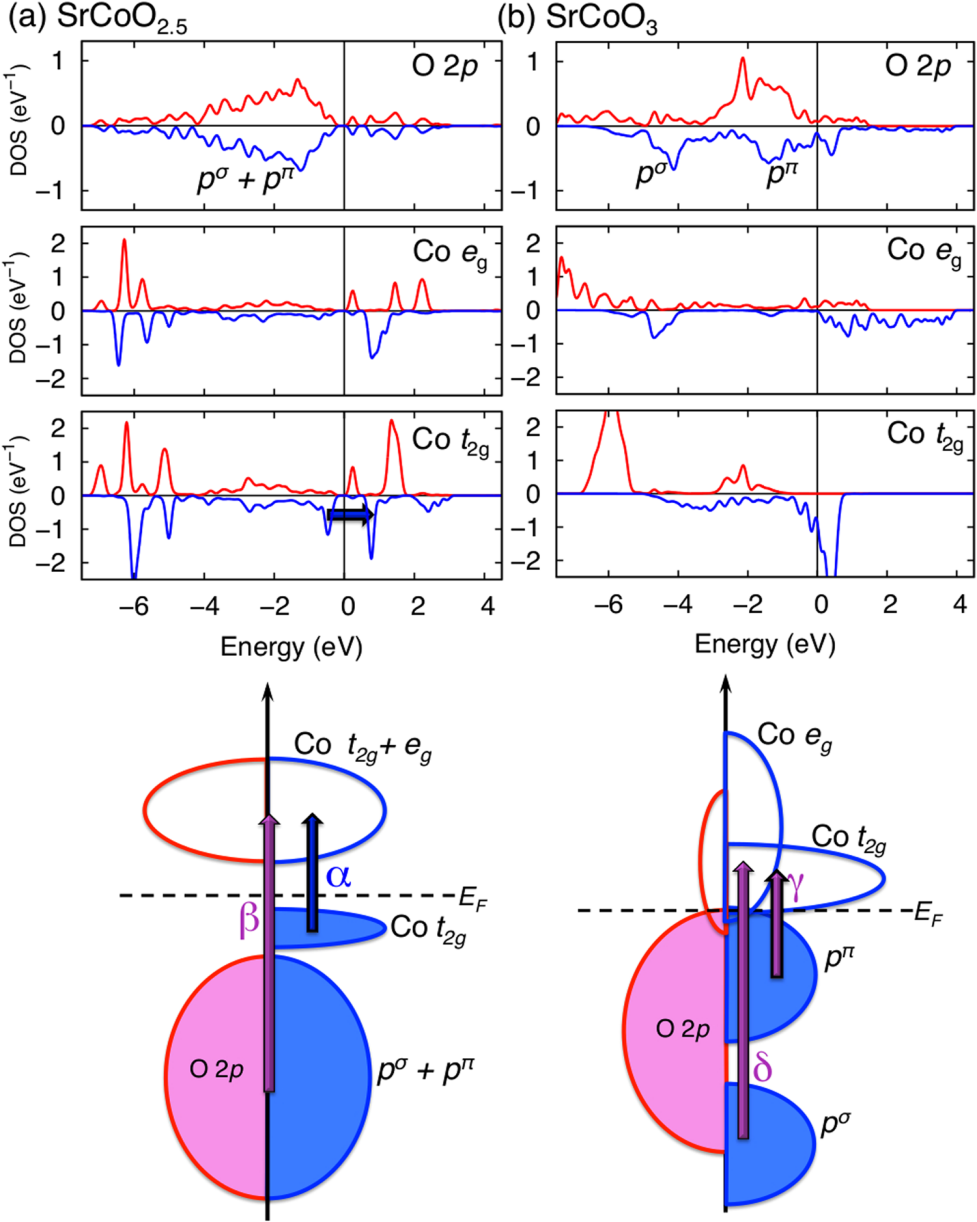
Electronic structure for BM- and P-SCO. Computed PDOS (eV^−1^) and electronic pictograms of (**a**) BM- (SrCoO_2.5_) and (**b**) P-SCO (SrCoO_3_). Blue and purple arrows denote *d-d* (Mott) and *p-d* (charge-transfer) excitations, respectively. A large charge-transfer excitation, *β*, occurs due to the orbital mixing in BM, while the excitation split into *γ* and *δ* due to the separation between *σ* and *π* of oxygen 2*p* orbitals. *p* ^*σ*^ and *p* ^*π*^ denote *σ* and *π* bonding of oxygen *p* orbitals. In BM-SCO, the overall *e*_g_ and *t*_2g_ distributions are rather similar because of mutual mixing induced by vacancy-induced lattice distortions. The electronic distribution becomes clearly separated in P-SCO due to the higher structural symmetry.

Figure 1 shows oxygen content (*x*) dependent optical conductivity spectra (*σ*_1_(*ω*)) computed using DFT calculations (Fig. 1(a)) and recorded by spectroscopic ellipsometry (Fig. 1(b)). The overall qualitative absorption features (*α*, *β*, *γ*, and *δ* peaks) are consistently revealed in both theoretical and experimental data. BM-SCO (see the spectrum for *x* = 2.5) shows an insulating behavior with two optical absorption peaks, *α* and *β*, representing Mott and charge-transfer gaps, respectively. Among them, *β* is the dominant absorption with a greater intensity. As shown in Fig. 2(a), the *β* peak shows possible transitions from oxygen 2*p* to Co 3*d* in BM phase. In the insulating phase, major magnetic exchange is super-exchange in the Co-oxygen-Co bridge, so the largest *β* peak from Co to oxygen shows a possible pathway in AFM super-exchange mechanism in BM phase.

With increasing *x*, as seen from data of both calculations and spectroscopic measurements in Fig. 1, the *β* peak splits into two peaks: *γ* and *δ*, due to the split of oxygen band under octahedral crystal field. The experimental data on MIX-SCO shows the transition, with a significant increase in the low lying absorption. This low-lying absorption might come from the Drude response which manifests the phase mixture between the PV- and BM-SCO. Alternatively, it might be the absorption at ~0.3 eV, shown in theoretical calculation (Fig. 1(a)). As shown in Fig. 2(b) for projected density of states (PDOS), *γ* and *δ* are optical transitions from *π* and *σ* characters of oxygen to Co 3*d* orbitals, respectively. Interestingly, the split arises concomitantly with the appearance of the Drude peak (see the data from increased *x* values), which represents the metallic character. Therefore, the split of the large insulating *β* peak into two major peaks (*γ* and *δ*) is strongly related to IMT with oxidation. The FM for highly oxidized SCO is induced by the itinerant hole conduction^22^, as the formation of *σ-*bond and subsequent straight bonding character of Co-O-Co are advantageous to the itinerancy and FM.

### Possibility of magnetically-driven IMT

In order to understand the main cause for IMT at *x* = 2.75, we comparatively calculated *σ*_1_(*ω*) of relaxed SrCoO_2.75_ for both FM and AFM configurations. The AFM configuration clearly shows an insulating optical spectrum [(see data marked *x* = 2.75 (AFM) in Fig. 1(a)] as for BM with the dominant *β* peak, whereas the *β* peak disappears with the emergence of Drude peak for FM configuration [(see data marked *x* = 2.75 (FM) in Fig. 1(a)] resembling that of P-SCO. We confirm that the Drude peak begins to appear from *x* ~2.75 in PV′-SCO (2.75 < *x* < 2.8) as in Fig. 1(b). Note that the oxidization state was confirmed by comparing x-ray absorption spectroscopy data with known spectra from bulk. This comparison clearly shows that electronic property, including insulating/metal character, is strongly subject to magnetic ordering; and metallic characters should be induced by the emergence of FM ordering at *x* ~2.75.

In particular, we find the *β* peak of our DFT calculations is about 1 eV higher than that of experiment. When we use smaller *U*, the difference gets smaller, which means the estimation of *p-d* transition is affected by the choice of *U*.

Figure 2 shows PDOS data calculated to explore the origin of optical absorption peaks. BM-SCO with 1D oxygen vacancy channels forms a fairly distorted structure and, thus, gives rise to an orbital mixing of *π* and *σ* bonds between oxygen and Co (Fig. 2(a)), which yields the strong *β* peak indicative of an insulating state. However, by filling the 1D oxygen vacancies with oxidation, the overall structure evolves into the cubic symmetry, and *σ*- and *π*-bonding are clearly separated. Consequently, *σ* bonding hybridization character is enhanced, so that the bandwidth of Co *e*_*g*_ spreads up to 4 eV as shown in Fig. 2(b). Therefore, a straighter and easier path for the hole of oxygen mediating Co spin is formed and induces Zener-type double exchange ferromagnetism. Distinct from other cubic perovskites (Sr*M*O_3_, *M* = V, Cr, Mn, and Fe), SrCoO_3_ is a unique FM, exhibiting a high Curie temperature (near room temperature), driven by the strong double exchange. The evolution from Co^3+^ to Co^4+^ lowers the electronic energy states, increasing the overlap with oxygen. This change may help sustain FM close to room temperature. Moreover, these coupled characters, i.e. the AFM super-exchange in insulator and the FM double exchange in metal, reinforce the possibility of the coupled magnetic IMT at around x = 2.75.

### Drastic transition

**Figure 3:**
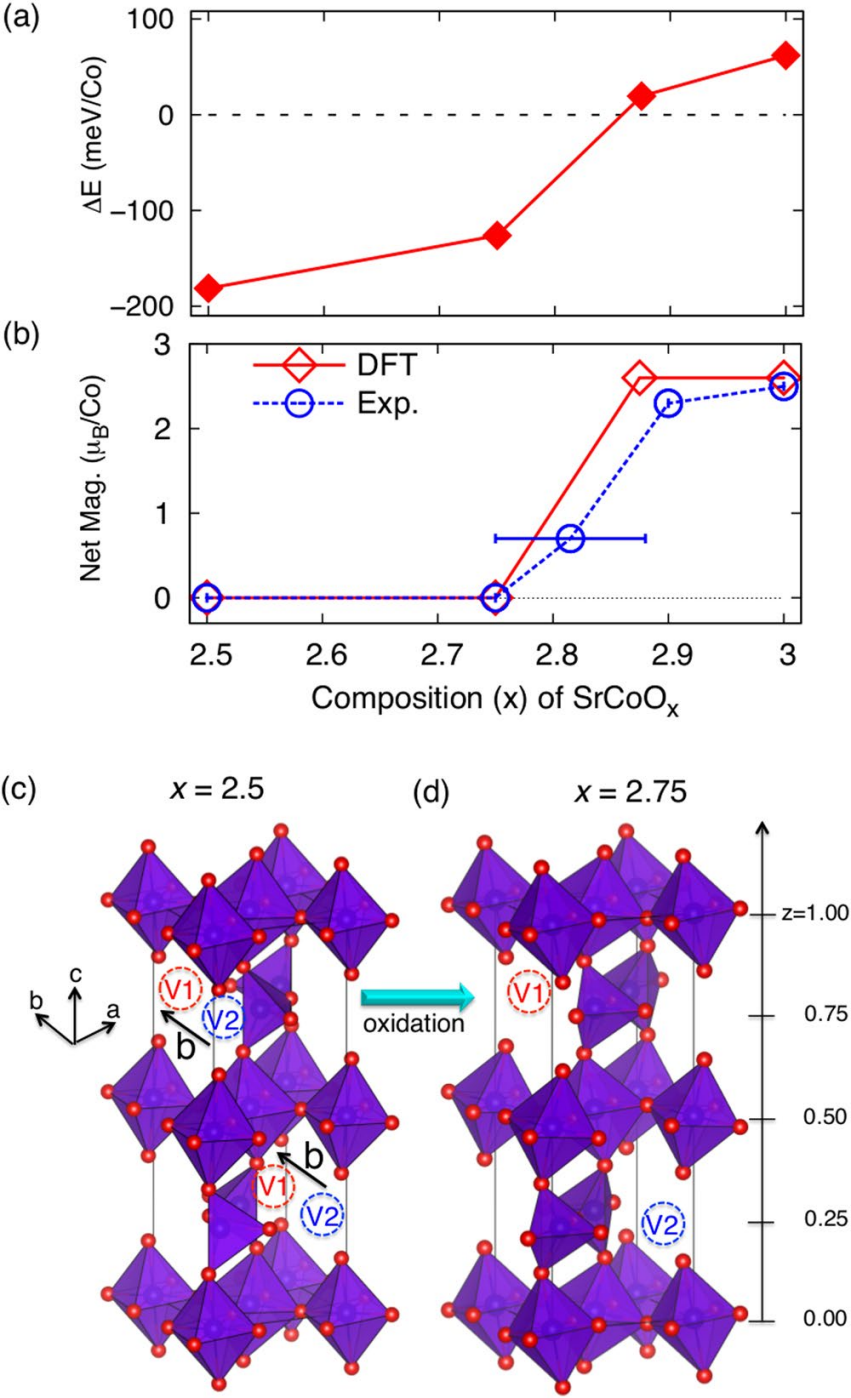
Rapid 1^st^-order-like phase transition at around x = 2.75 in SrCoO_*x*_. (**a**) Calculated energy difference (Δ*E* = *E*_AFM_ − *E*_FM_) between relaxed AFM and relaxed FM structures at given compositions (*x* = 2.5, 2.75, 2.875, and 3.0) (**b**) Net magnetic moments calculated from GGA + *U* (diamond, “DFT”) and compared with experiments (circle, “Exp.”) for *x* = 2.5 [ref.^10^], 2.75 [ref.^10^], 2.75~2.8 [ref.^32^], 2.9 [ref.^8^], and 3.0 [ref.^33^]. (**c**,**d**) Calculated lowest energy structure for SrCoO_2.5_ and SrCoO_2.75._ The V1 and V2 vacancies indicate different Wyckoff positions generated along the *b*-lattice within the *ab*-plane. Note that the distant vacancy sites are occupied with oxidation (V2 in *z* = 0.25 plane and V1 in *z* = 0.75 plane), so that the distance between the remnant oxygen vacancies in the lowest energy structure is maximized as shown in (**d**).

To check the character of the possibly coupled magnetic IMT, we carried out further DFT calculations and compared with experimental results as shown in Fig. 3. We used different compositions of SrCoO_*x*_ (*x* = 2.5, 2.75, 2.875, and 3.0) and in particular searched for different vacancy configurations for SrCoO_2.75_ and SrCoO_2.875_ to find the lowest energy structure. For every oxidation phase with the lowest energy, we calculated the energy difference between the G-AFM and FM relaxed phases as shown in Fig. 3(a). The energy difference shows an S-shape with a rather rapid increase around the transition (*x* = 2.75). It is worth mentioning that this rapidly increasing tendency from x = 2.75 does not change with a reasonable choice of U as we have tested, for instance, with *U*_eff_ = 2.5 eV. Moreover, as summarized in Fig. 3(b), we see the AFM-I phase is transformed to the FM-M phase at around 2.75. Therefore, a small modification in oxidation near x = 2.75 can induce a large change in electronic and magnetic properties. This transition can be further supported by a sudden increase in the *δ* peak upon oxidation (see Fig. 1), which is responsible for the increase in sigma bonding. Potze *et al*.^22^ proposed that an increase in sigma bonding can induce the double exchange interaction, resulting in enhanced FM^21^. Consistently, we previously observed coexistence of the two phases, i.e. BM-SCO and PV-SCO, with an increased FM response upon oxidation without any intermediate phase^10^. As shown in Table 1, increase in bond-angle of Co-O-Co induces formation of the straight *σ*–bond that drives the concurrent metallicity.

**Table 1:** Bond angle (Co-O-Co) information (°) calculated from DFT.

	x = 2.5			x = 2.75		x = 2.875		x = 3.0	
	AFM	FM	AFM		FM	AFM	FM	AFM	FM
In-plane	132,173	129,172	164,178		168,178	170,177	172,178	180	180
Out-of-plane	150	147	155,160		155,162	165,168	161,173		

We further compared the calculated magnetic moments with the experimental values. The change in experimental magnetic moments shown in Fig. 3(b) is in good agreement with the theoretical results, showing a rather rapid increase at around *x* = 2.75. A magnetic transition is known to occur at around *x* = 2.75 and MIT at *x* = 2.9^24^. Figure 3(c) and (d) show stable vacancy positions at *x* = 2.5 and *x* = 2.75. Based on the calculated structures, we conjecture that oxidation from x = 2.5 to x = 2.75 fill V1 in *z* = 0.25 plane and V2 in *z* = 0.75 plane, the most distant vacancies in the BM unit-cell. The simultaneous filling of the distant oxygen vacancy sites circumvents vacancy clustering so is advantageous to fast and effective oxidation. The measured magnetic moments of Co1 and Co3 sites of AFM BM phase are about 3.1 μ_B_/Co and 2.9 μ_B_/Co (Fig. 9 of ref.^25^), respectively. Our calculated moments are 2.9 μ_B_/Co and 2.8 μ_B_/Co, respectively.

### Magnetically-driven metallic state

**Figure 4:**
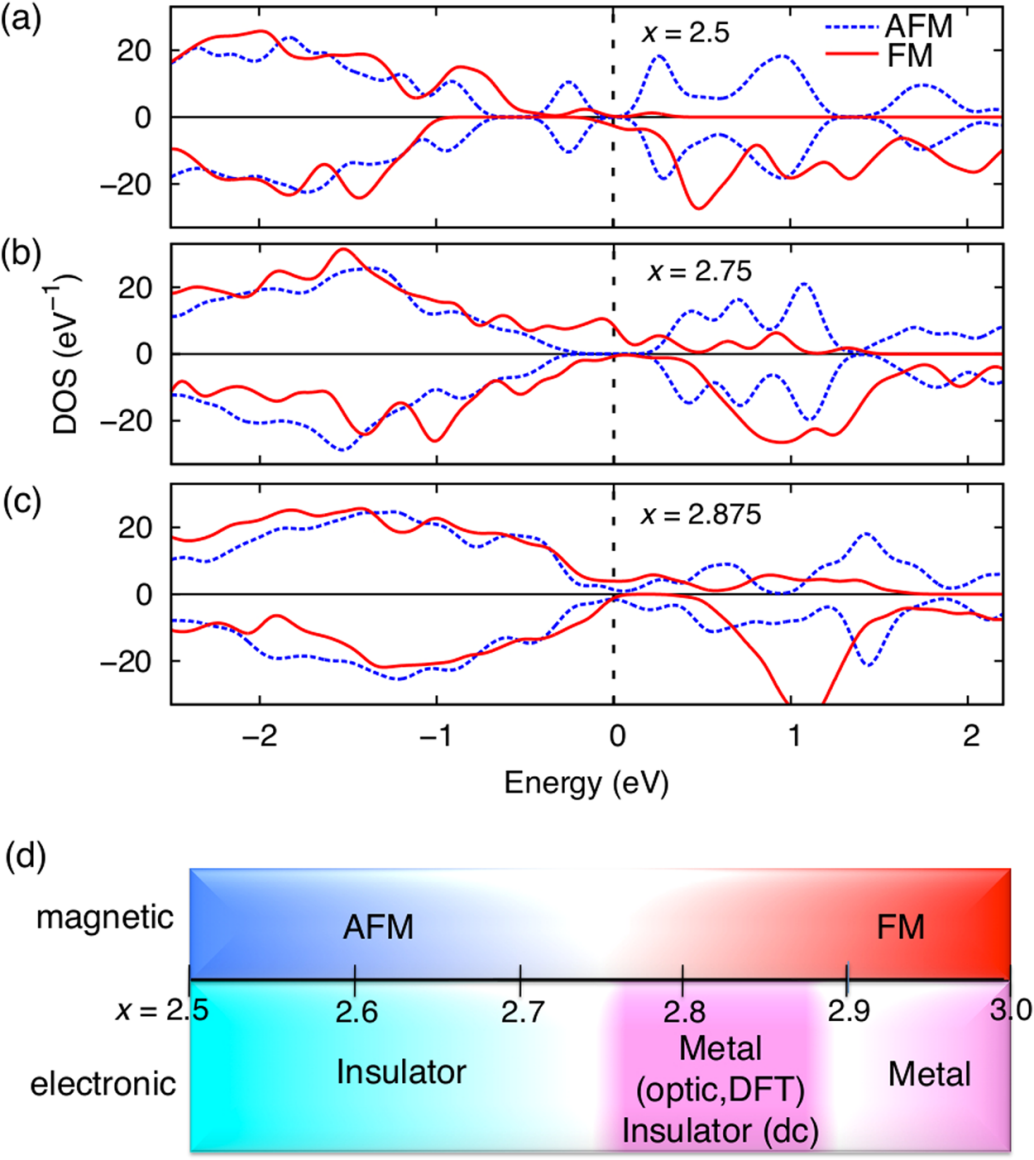
Coupled magnetic and electronic phase transitions. Total DOS of AFM (dotted line) and FM (bold line) relaxed structures for *x* =  (**a**) 2.5, (**b**) 2.75, and (**c**) 2.875. Note that FM always induces metallic characters regardless of the composition. (**d**) Expected phase diagram of SCO with oxidation from our optical study combined with DFT. It is compared with a previous dc-measurement^24^.

Figure 4 shows total DOS for BM and two intermediates (*x* = 2.75 and 2.875) close to the transition. We computed for both AFM and FM phases to check the coupling of magnetism and metallicity. In SrCoO_*x*_ (*x* = 2.5~2.875), the metallic character always appears with FM ordering at the intermediate phases, whereas they are insulating with AFM at 2.5 and 2.75. The proposed double exchange interaction^22^ can be facilitated with the structure starting at *x* = 2.75 with oxidation^23^. In Fig. 4(d), we show a schematic of the phase diagram from our optical measurements and DFT results compared with the previous *dc*-measurements^24^. Although our combined optical study with DFT calculations shows a coupled magnetic IMT at *x* = 2.75, the *dc* transport measurement shows the IMT at around *x* = 2.9. Thus, this offset of the IMT could be associated with extrinsic effects, such as phase separation by forming puddles of conducting charges, domain boundaries, etc., which can reduce electrical conductivity yielding poor electronic conduction in *dc*-measurements. Similar discrepancy between the optical study and *dc*-measurement was reported in colossal magnetoresistive (CMR) (La,Sr)MnO_3_^26^.

## Conclusions

In summary, we provide experimental and computational evidence that FM transition induces concurrent IMT in SrCoO_*x*_. First, the room-*T* AFM to high-*T* FM transition is a rare magnetic transition, so that such a distinct transition requires additional driving force such as IMT as happened in CMR. Also, a rather rapid change is found in magnetic energy and moment at around *x* = 2.75, which may be affected by the IMT. Second, our DFT study reveals that formation of the straight *σ*–bond drives the concurrent metallicity. Thus, the orthorhombic to cubic transition at *x* = 2.75 (ref.^23^) forming straight bonds can drive the metallicity at the same composition. Lastly, our DFT results confirm that the metallic state is always accompanied by FM ordering regardless of the degree of oxidation in SrCoO_*x*_, so the FM transition at *x* = 2.75 should trigger IMT. We further note that IMT in TMOs has been recognized as an important ingredient for many technological applications. We show that oxygen intercalation, which is known to occur as low as 200 °C in SrCoO_*x*_ oxygen sponges can be a powerful means to induce IMT without complicated chemical doping of cations. This vacancy-induced, magnetically coupled IMT can be compared with pressure-induced, magnetically coupled IMT in rare-earth double perovskites^27^. It is also worth noting that TMOs have many order parameters, including spin, charge, lattice, and orbital. Since the strong interactions among the order parameters can be delicately controlled by oxygen content, the fundamental understanding of the coupling between the magnetic and electronic properties reported here will bring a tremendous impact on tailoring diverse functionalities.

## Methods

### First-principles calculations

DFT calculations were performed within the generalized gradient approximation GGA + *U* method with the Perdew-Becke-Erzenhof parameterization as implemented in the Vienna ab initio Simulation Package (VASP-5.2)^28^. The Projector augmented wave (PAW) potentials^29^ include ten valence electrons for Sr (4*s* ^2^4*p* ^6^5*s* ^2^), nine for Co (3*d* ^8^4*s* ^1^), and six for oxygen (2*s* ^2^2*p* ^4^). The wave functions are expanded in a plane wave basis with 500 eV energy cutoff. For BM-SCO, a 3 × 1 × 3 Monkhorst-Pack *k*-point grid was used for relaxation and 6 × 2 × 6 grid was used for optical property and density of states. For P-SCO, a 14 × 14 × 14 grid was used. We use the Dudarev implementation^30^ with on-site Coulomb interaction *U* = 4.5 eV and on-site exchange interaction *J*_H_ = 1.0 eV to treat the localized *d* electron states in Co with *U*_eff_ = 3.5 eV. A similar choice of *U* was successfully applied to BM-SCO for analysis of its electronic and magnetic properties^31^. To find the minimum energy configuration in the given compositions (x = 2.5, 2.75, and 2.875), we used a $\sqrt{2}\times 4\times \sqrt{2}$ super cell (40 atoms). For SrCoO_2.75_, we compared total energies of different vacancy configuration and chose the lowest energy configuration where the two oxygen vacancies are most distant in unit-cell. The calculated lattice parameters are shown in Table 2.

**Table 2:** Lattice information calculated from DFT.

	x = 2.5		x = 2.75		x = 2.875		x = 3.0	
	AFM	FM	AFM	FM	AFM	FM	AFM	FM
*c*-lattice (Å)	5.746	5.804	5.627	5.616	5.591	5.585	5.527	5.384

*c*-lattice parameters of calculated SrCoO_*x*_ (*x* = 2.5, 2.75, 2.875, 3.0) epitaxially-strained on LSAT substrate (*a* = *b* = 3.87 Å).

Experimental intensity is lower than theoretical one because extrinsic effects in experimental conditions may reduce the optical conductivity. Also, positions of the frequency (ω) values in the theoretical peak depend on the choice of *U*. Therefore, a smaller *U* may shift lower the peaks and gives rise to better agreement with experimental results.

### Sample preparation and spectroscopic ellipsometry

We used pulsed laser epitaxy to grow epitaxially strained SCO thin films on (001) (LaAlO_3_)_0.3_(SrAl_0.5_Ta_0.5_O_3_)_0.7_ (LSAT) substrates. We kept the sample thickness as 55 nm. Different annealing conditions were applied to systematically change the oxidation state of the BM-SCO thin films. P-SCO was also directly grown in O_2_ + O_3_ (5%) environment. Detailed sample preparation and other properties can be found elsewhere^8,10^. Spectroscopic ellipsometry was performed using an ellipsometer (M-2000, J. A. Woollam Co., Inc.) between 0.4 and 5.4 eV at an incident angle of ~60°, 70°, and 80°. Simple two-layer (film/substrate) model fit was used to successfully deduce the complex dielectric functions of thin films.
